# Principles of Lifeworld Led Public Health Practice in the UK and Sweden: Reducing Health Inequalities

**DOI:** 10.1155/2015/124591

**Published:** 2015-01-06

**Authors:** Ann Hemingway, Liz Norton, Clara Aarts

**Affiliations:** ^1^Faculty of Health and Social Sciences, Bournemouth University, Poole, UK; ^2^Uppsala University, 751 05 Uppsala, Sweden

## Abstract

The purpose of this paper is to consider the role of the lifeworld perspective in reducing inequalities in health and we explain how the public health practitioner can use this perspective to address public health issues with individuals and groups. We offer ideas for public health actions that are based on and deal with the lifeworld context of individual people or families. Each of the dimensions of the lifeworld temporality, spatiality, intersubjectivity, embodiment and mood are outlined and their significance explained in relation to health inequalities. Suggestions for action to reduce health inequalities are made and overall principles of lifeworld led public health practice are proposed by way of conclusion. The principles comprise understanding the community members' lifeworld view, understanding their view of their potential, offering resources and facilitating empowerment, and sharing lifeworld case studies and lobbying to influence local and national policy in relation to both the individual and communities.

## 1. Introduction

This paper will consider the question of how the public health practitioner works to reduce health inequalities for individuals and groups in their practice context. A suggestion is that public health practitioners should not only be aware of the vulnerabilities or needs of the people in their areas of practice but also be familiar with their “clients” perspectives of their own worlds; that is, practitioners should consider a lifeworld led approach to their practice. Recently in the UK, Public Health England (PHE) has advocated that the “lived experience” of people in communities needs to be gleaned in order for health inequalities to be addressed effectively [1]. This is because we are stakeholders in our communities and more than this we have the essential real-life experience of living in our community, our world. Lifeworld led research uses a phenomenological approach to understand the lived experience of people therefore helping practitioners to understand what it means to live within a particular context and community. What this means as well as how it can be used as an approach to inform practice to reduce health inequalities is discussed next.

Inequalities in health are “Differences in the prevalence or incidence of health problems between individual people of higher and lower socio-economic status” [2]. In the UK Public Health England publish health profiles annually. This is also the case in Sweden. These profiles give information about the health issues affecting communities and are targeted at local public health practitioners. The information is intended as a resource to inform health planning and to reduce health inequalities. The profiles compare local health indicators with those nationally and within the specified area [3]. Although a geographical area may fare better than the national average overall the health profiles can also indicate pockets of deprivation and health inequality.

## 2. Inequalities in Health in the UK and Sweden

The latest Annual Health Report in Sweden [4] describes positive health development for the population as a whole. However there are still inequalities in health, some of which are increasing. For example, in recent years, the rise in milder mental health problems appears to have ceased, but the smaller proportion of people who experience severe apprehension, agitation, or anxiety has continued to increase particularly among young people. Differences between groups with differing educational backgrounds are still evident. All the major causes of death in the population, cardiovascular disease, stroke, cancer, accidents, suicide, and alcohol-related illnesses are more common among those with less education. This group also reports a worse general state of health and has more mental health issues.

Inequalities in health in the UK are widespread across the country and well established [5]. In absolute terms, the poor have become worse off in recent years, particularly when their housing costs are properly accounted for. Important new themes have emerged, including the falling-behind of young adults as they have struggled in the labour market and large differences in trends in the cost of housing across the population as mortgage interest rates have plummeted [6]. The absolute inequality in mortality between the Higher Managerial and Professional class (most advantaged) and the Routine class (least advantaged) narrowed over time, but the relative inequality increased, for both sexes [7].

There is a wider tradition of thought about how we act in our world and this paper suggests that there is a need for public health as a discipline to move outside the more mechanical or reductionist biological model of human meaning and behavior to address the complex issue of the reduction of inequalities in health [8]. This may require us to consider our intersubjective world, or how we are in our social world with others in order to understand behavior. The lifeworld engages individual, social, perceptual, and practical experience; it is where reflection takes place [9].

The purpose of this paper is to introduce the practitioner to the concept of lifeworld and to allow its translation into practice. The authors acknowledge that there is a risk of over simplification of the philosophical background to this concept; however we feel it is important to share preliminary ideas about how this concept can be applied in practice. For this reason the authors have drawn on the contemporary work of Galvin and Todres [10] who have applied this concept to health care.

## 3. The Elements of the Lifeworld

The “lifeworld” view has emerged from the work of philosophers such as Husserl [9], Merleau-Ponty [11], and Habermas [12]. Husserl was a mathematician who became concerned in relation to the limitations of quantitative measures in relation to the human experience. Five elements of “lifeworld” have emerged from this historical body of work. These are temporality, spatiality, intersubjectivity, embodiment, and mood [9, 10]. Todres et al. [13] have highlighted how we are humans in a holistic context that incorporates dimensions of lifeworld and Hemingway [14] has outlined our lifeworld can impact on our wellbeing as it changes and is arguably manipulated by outside influences such as political policy [15]. The dimensions of lifeworld are outlined below and their significance in terms of public health practice and the reduction of inequalities in health is explained.

## 4. Temporality

Temporality refers to time as experienced by human beings, for example, the rhythm of the seasons. As we as human beings adapt to the pressures of our time in a horological sense this can have a negative impact on our health and wellbeing and become an unmanageable pressure. This can limit our potential and prevent us from seeking out and experiencing new possibilities. Not having enough time or indeed having too much time to fill can be perceived as stressful and does indeed have a negative impact on our health. The recent report in the UK by the All-Party Parliamentary Group on Wellbeing Economics [1] cites evidence that unemployment impacts wellbeing in a negative way which is far more extensive than the impact of lost income alone. While there is evidence that staff working unsocial hours have lower job satisfaction and motivation which results in dissatisfaction with life in general, loss of wellbeing and quality of life, and poor performance [16, 17]. While long and unpredictable hours can lead to mental stress [18].

The significance of the above in relation to temporality is that feelings of possibility relate to our temporal experience of the world. They can emerge through thoughts about personal histories and memories or indeed from our aspirations for the future [13]. However, time can also be viewed as an asset: how can the individual or family see their time in this way and how can we as public health practitioners help support this view? We could explore participation, occupation, and engagement in relation to the lifeworld context of the individual in their context and help to influence local policy strategically and in the more personal context offer ideas or opportunities. In addition there may be scope to influence national policy, for example, regarding the length of the working day or week or legislation in relation to flexible working.

## 5. Spatiality

Spatiality refers to our human environmental context and our experience of living in that environment. The way we interact with our environment and the qualities of that environment can have a positive or negative impact on wellbeing [14]. Our own personal “geography” can impact on our wellbeing or health behaviour or put our personal safety at risk, just as it can also promote health. Our personal environments can offer opportunities for socialization and meaningful activity, or access to the natural world's aesthetics along with arts and sport for instance. These factors can all improve our feelings of wellbeing [19]. Conversely our personal environments may not offer these opportunities. This could be seen as a continuum of opportunity in relation to public health practice, with one end of this continuum relating to someone who is homeless or a refugee whose ability to feel “at home” and secure in their environment is lost and includes living in a resource poor area right through to the other end of the continuum where the opportunities outlined above are readily available. In terms of public health practice it is crucial to understand the lifeworld of people and their potential issues in relation to the public health spatiality continuum outlined here. On an individual basis this may mean helping with access to accommodation, services, and social support. It may also mean sharing lifeworld experiences to ensure adequate resources and support are available. This may be achieved through enlightening local politicians and multidisciplinary practitioners in relation to the real world for those experiencing it and in so doing influence local policy.

## 6. Intersubjectivity

Through intersubjectivity we make sense of our interpersonal world and others who share it. This intersubjectivity allows us to frame our thinking, our identity, and our relationships in time and space. For example, it can be reflected in our consideration of who we are close to, who we love, or who we want to be with. Intersubjectivity also helps us navigate our cultural and traditional contexts which impacts on our self-perception and how we view others [14]. In terms of public health practice this relates to individuals and family interrelationships and their impact on health and wellbeing. Intersubjectivity may relate to positive and negative experiences such as domestic violence, social isolation, or positive supportive relationships and communities. The implications for the public health practitioner are that through understanding peoples lifeworld contexts we are more able to support them in a way which is culturally acceptable to them while still aiming at maintaining their safety and beneficial long term relationships. In relation to local policy this may mean lobbying for appropriate accessible support through sharing lifeworld case studies and empowering individuals to share their experiences as they feel comfortable with.

## 7. Embodiment

Being human, we live within our bodies and experience the world through them [12]. Embodiment has been articulated as a key concept within an ecological perspective on public health with human beings being perceived as holistic entities or beings in terms of their biology and sociological and physical environment [20–22]. It is seen as the means by which humans biologically integrate the physical and social environment they inhabit throughout their lives. Embodiment means that one's biology cannot be understood without considering psychosocial and sociocultural aspects of an individual's development [23]. Embodiment relates to how we experience the world including our perceptions of our context and its possibilities or limits as experienced through our bodies [14].

We would argue that low birth weight babies and obesity rates which are higher in low income groups in both the UK and Sweden [2, 4] reflect the embodiment of health inequalities. Understanding the lifeworld context of people's health behaviour is crucial to enabling behaviour change effectively. Offering support and advice which does not make sense to the individual's context or life view is meaningless [8]; hence, commissioning as well as providing meaningful support is crucial as suggested through the concept of social marketing [24]. In addition public health practitioners are responsible for influencing local policy to ensure appropriate support and partnership working occurs to support behaviour change. For instance, the lived experience of behaviour change in relation to taking more exercise in an area where personal safety on the street is an issue or being unable to access sports facilities due to the cost. Public health practitioners need to work not only to enable behaviour change but also to positively impact the environment in which that change takes place. This may be achieved by lobbying at a local or national level.

## 8. Mood

The lived experience is hugely influenced by mood which also helps shape the other dimensions of the lifeworld [13]. It will influence and impact on our physical and mental wellbeing and crucially is influenced by the other dimensions outlined above. Mood is an essential element of how we are as human beings and affects our ability to realize our potential. For example, anxiety reveals a very different lifeworld than joy and sorrow [14]. Rates of suicide and depression are on the increase in Europe despite economic growth. The World Health Organisation [25] has predicted that depression (as a gross measurement of wellbeing) if unchecked will soon be a leading global cause of disability. This increase may be due in part to improved diagnosis; however overall it appears that “consumerism” is not having a positive impact on our mental wellbeing [26].

In relation to public health practice and the reduction of inequalities in health a lifeworld led approach enables insight into the potential negative impacts of ones circumstances in relation to mood and what factors impact on mood for that individual or family. This would help to plan appropriate support and interventions and would also give insight to local policy makers regarding how to offer support and developments which support mental health and wellbeing in an area.

## 9. Conclusion

This paper has outlined the elements of the lifeworld and their relevance for public health practice in relation to the reduction of health inequalities. There are some common principles which have emerged through the consideration of these elements in both the UK and Sweden which help to inform lifeworld led public health practice:

1. understand the person's lifeworld by listening to their view of their current situation and their potential, in relation to
  - participation, occupation, engagement, and income (temporality),
  - the public health spatiality continuum (spatiality),
  - safety and relationships (intersubjectivity),
  - the context and potential for health behaviour change (embodiment),
  - what factors may impact on mental health and wellbeing for a particular individual or family (mood);
2. offer resources and empowerment based support as appropriate;
3. share and lobby using lifeworld led case studies and enable community members to influence local and national policy and partnership working in relation to the individual, their family, and their local communities.
